# Supplementary figures and images for: High-frequency terahertz stimulation alleviates neuropathic pain by inhibiting the pyramidal neuron activity in the anterior cingulate cortex of mice
Source: eLife. 2024 Sep 27;13:RP97444. doi: 10.7554/eLife.97444 (PMC11434610; doi:10.7554/eLife.97444)

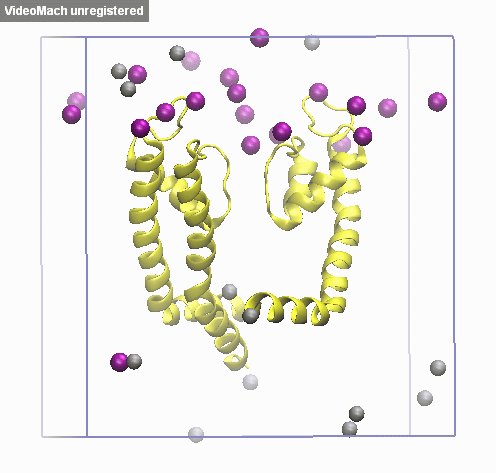

Supplement: Figure 1—source data 1. [file elife-97444-fig1-data1.zip › Fig. 1/K-channel-HFTS.gif]

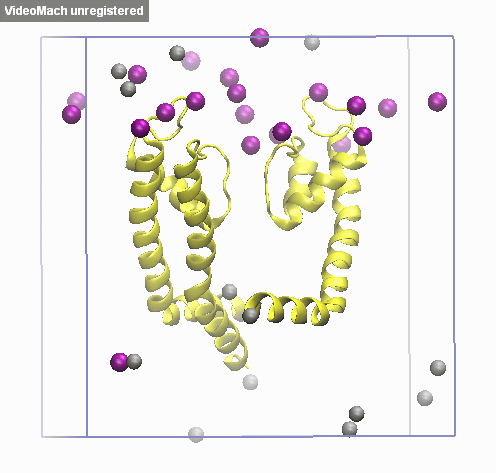

Supplement: Figure 1—source data 1. [file elife-97444-fig1-data1.zip › Fig. 1/K-channel-Without HFTS.gif]

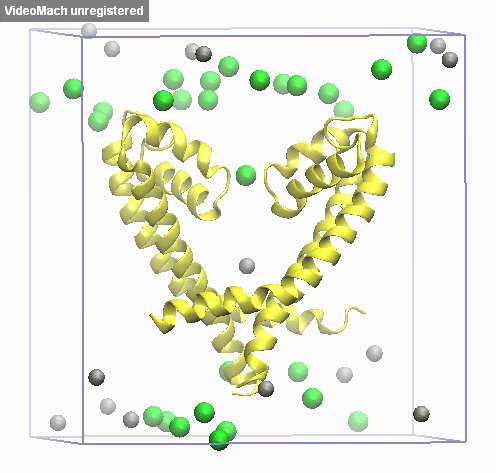

Supplement: Figure 1—source data 1. [file elife-97444-fig1-data1.zip › Fig. 1/Na-channel.gif]

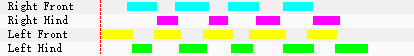

Supplement: Figure 5—source data 1. [file elife-97444-fig5-data1.zip › Fig. 5/SHAM/SAHM_2.png]

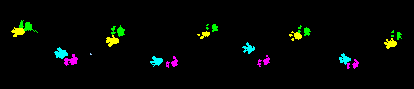

Supplement: Figure 5—source data 1. [file elife-97444-fig5-data1.zip › Fig. 5/SHAM/SHAM_1.png]

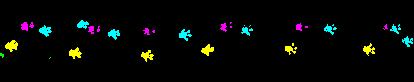

Supplement: Figure 5—source data 1. [file elife-97444-fig5-data1.zip › Fig. 5/SNI/SNI_1.png]

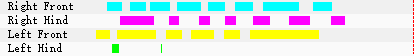

Supplement: Figure 5—source data 1. [file elife-97444-fig5-data1.zip › Fig. 5/SNI/SNI_2.png]

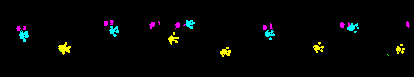

Supplement: Figure 5—source data 1. [file elife-97444-fig5-data1.zip › Fig. 5/SNI+BLS/SNI+BLS_1.png]

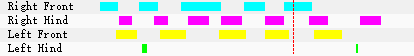

Supplement: Figure 5—source data 1. [file elife-97444-fig5-data1.zip › Fig. 5/SNI+BLS/SNI+BLS_2.png]

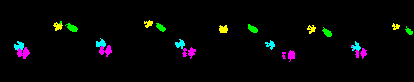

Supplement: Figure 5—source data 1. [file elife-97444-fig5-data1.zip › Fig. 5/SNI+HFTS/SNI+HFTS_1.png]

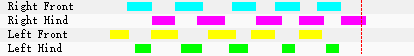

Supplement: Figure 5—source data 1. [file elife-97444-fig5-data1.zip › Fig. 5/SNI+HFTS/SNI+HFTS_2.png]
