# Supplementary material for: High-frequency terahertz stimulation alleviates neuropathic pain by inhibiting the pyramidal neuron activity in the anterior cingulate cortex of mice
Source: eLife. 2024 Sep 27;13:RP97444. doi: 10.7554/eLife.97444 (PMC11434610; doi:10.7554/eLife.97444)
Supplement: Supplementary file 1. — (a) The Kv current to different voltage in PYR of SNI mice before and after HFTS. (b) Changes of the Kv current impact by HFTS over time. (c) The Kleakcurrent to different voltage in PYR of SNI mice before and after HFTS [file elife-97444-supp1.docx]

| Supplementary file 1a. The Kv current to different voltage in PYR of SNI mice before and after HFTS | | | | | |
| --- | --- | --- | --- | --- | --- |
| SNI - SNI+HFTS | Mean Diff. | 95.00% CI of diff. | Below threshold? | Summary | Adjusted P Value |
| -110 mV | 35.81 | -1266 to 1338 | No | ns | >0.9999 |
| -100 mV | 91.01 | -1211 to 1393 | No | ns | >0.9999 |
| -90 mV | 51.45 | -1250 to 1353 | No | ns | >0.9999 |
| -80 mV | -1.271 | -1303 to 1301 | No | ns | >0.9999 |
| -70 mV | 37.7 | -1264 to 1339 | No | ns | >0.9999 |
| -60 mV | 21.7 | -1280 to 1323 | No | ns | >0.9999 |
| -50 mV | 35.37 | -1266 to 1337 | No | ns | >0.9999 |
| -40 mV | 58.42 | -1243 to 1360 | No | ns | >0.9999 |
| -30 mV | 159.3 | -1142 to 1461 | No | ns | >0.9999 |
| -20 mV | 208.7 | -1093 to 1510 | No | ns | >0.9999 |
| -10 mV | -25.13 | -1327 to 1277 | No | ns | >0.9999 |
| 0 mV | -98.64 | -1400 to 1203 | No | ns | >0.9999 |
| 10 mV | -529.7 | -1832 to 772.0 | No | ns | 0.9969 |
| 20 mV | -830.9 | -2133 to 470.9 | No | ns | 0.7063 |
| 30 mV | -1010 | -2311 to 292.1 | No | ns | 0.3384 |
| 40 mV | -1294 | -2596 to 7.778 | No | ns | 0.053 |
| 50 mV | -1471 | -2773 to -169.7 | Yes | * | 0.0127 |
| 60 mV | -1726 | -3028 to -424.5 | Yes | ** | 0.0012 |
| 70 mV | -1816 | -3118 to -514.3 | Yes | *** | 0.0005 |
| 80 mV | -1856 | -3157 to -553.8 | Yes | *** | 0.0003 |
| 90 mV | -1900 | -3202 to -598.7 | Yes | *** | 0.0002 |
| 100 mV | -1839 | -3141 to -537.0 | Yes | *** | 0.0004 |
| 110 mV | -1915 | -3216 to -612.7 | Yes | *** | 0.0002 |
| 120 mV | -1912 | -3214 to -610.4 | Yes | *** | 0.0002 |
| 130 mV | -1912 | -3214 to -610.0 | Yes | *** | 0.0002 |
| ANOVA table | SS | DF | MS | F (DFn, DFd) | P value |
| Row Factor x Column Factor | 55521178 | 24 | 2313382 | F (24, 240) = 8.581 | P<0.0001 |
| Row Factor | 511369305 | 24 | 21307054 | F (24, 240) = 79.03 | P<0.0001 |
| Column Factor | 45335332 | 1 | 45335332 | F (1, 10) = 6.846 | P=0.0258 |
| Subject | 66223673 | 10 | 6622367 | F (10, 240) = 24.56 | P<0.0001 |
| Residual | 64706213 | 240 | 269609 |  |  |
| Data summary | Šídák's multiple comparisons test | | | | |
| Number of columns (Column Factor) | 2 | | | | |
| Number of rows (Row Factor) | 25 | | | | |
| Number of subjects (Subject) | 12 | | | | |
| Number of missing values | 0 | | | | |

| Supplementary file 1b. Changes of the Kv current impact by HFTS over time | | | | | |
| --- | --- | --- | --- | --- | --- |
|  | Mean Diff. | 95.00% CI of diff. | Below threshold? | Summary | Adjusted P Value |
| Pre HFTS vs. 5 min | -899.9 | -1611 to -188.4 | Yes | * | 0.0103 |
| Pre HFTS vs. 20 min | -790.3 | -1502 to -78.79 | Yes | * | 0.026 |
| 5 min vs. 20min | 109.6 | -601.9 to 821.1 | No | ns | 0.9953 |
| ANOVA table | SS | DF | MS | F (DFn, DFd) | P value |
| Treatment (between columns) | 1969090 | 4 | 492273 | F (4, 15) = 4.193 | P=0.0178 |
| Residual (within columns) | 1761043 | 15 | 117403 |  |  |
| Total | 3730134 | 19 |  |  |  |
| Data summary | Šídák's multiple comparisons test | | | | |
| Number of treatments (columns) | 5 | | | | |
| Number of values (total) | 20 | | | | |

| Supplementary file 1c. The K_leak_ current to different voltage in PYR of SNI mice before and after HFTS | | | | | |
| --- | --- | --- | --- | --- | --- |
| SNI - SNI+HFTS | Mean Diff. | 95.00% CI of diff. | Below threshold? | Summary | Adjusted P Value |
| -120 mV | 19.22 | -2.693 to 41.14 | No | ns | 0.129 |
| -110 mV | 14.26 | -7.660 to 36.17 | No | ns | 0.4949 |
| -100 mV | 11.54 | -10.37 to 33.46 | No | ns | 0.7675 |
| -90 mV | 6.985 | -14.93 to 28.90 | No | ns | 0.9894 |
| -80 mV | 5.231 | -16.68 to 27.15 | No | ns | 0.999 |
| -70 mV | -1.408 | -23.32 to 20.51 | No | ns | >0.9999 |
| -60 mV | -7.848 | -29.76 to 14.07 | No | ns | 0.9752 |
| -50 mV | -14.48 | -36.40 to 7.432 | No | ns | 0.472 |
| -40 mV | -30.52 | -52.43 to -8.600 | Yes | ** | 0.0012 |
| -30 mV | -46.87 | -68.79 to -24.95 | Yes | **** | <0.0001 |
| ANOVA table | SS | DF | MS | F (DFn, DFd) | P value |
| Row Factor x Column Factor | 13968 | 9 | 1552 | F (9, 108) = 8.377 | P<0.0001 |
| Row Factor | 122682 | 9 | 13631 | F (9, 108) = 73.58 | P<0.0001 |
| Column Factor | 674.2 | 1 | 674.2 | F (1, 12) = 1.688 | P=0.2182 |
| Subject | 4792 | 12 | 399.3 | F (12, 108) = 2.156 | P=0.0189 |
| Residual | 20008 | 108 | 185.3 |  |  |
| Data summary | Šídák's multiple comparisons test | | | | |
| Number of columns (Column Factor) | 2 | | | | |
| Number of rows (Row Factor) | 10 | | | | |
| Number of subjects (Subject) | 14 | | | | |
| Number of missing values | 0 | | | | |
