# Supplementary material for: High-frequency terahertz stimulation alleviates neuropathic pain by inhibiting the pyramidal neuron activity in the anterior cingulate cortex of mice
Source: eLife. 2024 Sep 27;13:RP97444. doi: 10.7554/eLife.97444 (PMC11434610; doi:10.7554/eLife.97444)
Supplement: Supplementary file 2. — (a) The spike frequency in pyramidal (PYR) of anterior cingulate cortex (ACC) before and after spared nerve injury (SNI). (b) The spike frequency in PYR of SNI mice before and after high-frequency terahertz stimulation (HFTS). (c) The spike frequency in PYR of spared nerve injury (SNI) mice before and after blue light stimulation (BLS). (d) The spike frequency in PYR of Sham mice before and after HFTS [file elife-97444-supp2.docx]

| Supplementary file 2a. The spike frequency in PYR of ACC before and after SNI | | | | | |
| --- | --- | --- | --- | --- | --- |
| Table Analyzed | Sham vs SNI | | | | |
| Two-way RM ANOVA | Matching: Across row | | | | |
| Assume sphericity? | Yes | | | | |
| Alpha | 0.05 | | | | |
| Source of Variation | % of total variation | P value | P value summary | Significant? |  |
| Row Factor x Column Factor | 1.652 | 0.1771 | ns | No |  |
| **Row Factor** | **54.75** | **<0.0001** | ******** | **Yes** |  |
| **Column Factor** | **18.86** | **<0.0001** | ******** | **Yes** |  |
| Subject | 18.67 | 0.0003 | *** | Yes |  |
| ANOVA table | SS | DF | MS | F (DFn, DFd) | P value |
| Row Factor x Column Factor | 121.6 | 7 | 17.37 | F (7, 40) = 1.555 | P=0.1771 |
| Row Factor | 4030 | 7 | 575.7 | F (7, 40) = 16.76 | P<0.0001 |
| Column Factor | 1388 | 1 | 1388 | F (1, 40) = 124.2 | P<0.0001 |
| Subject | 1374 | 40 | 34.35 | F (40, 40) = 3.075 | P=0.0003 |
| Residual | 446.9 | 40 | 11.17 |  |  |
| Difference between column means |  | | | | |
| Mean of Con | 9.792 | | | | |
| Mean of SNI | 17.4 | | | | |
| Difference between means | -7.604 | | | | |
| SE of difference | 0.6823 | | | | |
| 95% CI of difference | -8.983 to -6.225 | | | | |
| Data summary |  | | | | |
| Number of columns (Column Factor) | 2 | | | | |
| Number of rows (Row Factor) | 8 | | | | |
| Number of subjects (Subject) | 48 | | | | |
| Number of missing values | 0 | | | | |

| Supplementary file 2b. The spike frequency in PYR of SNI mice before and after HFTS | | | | | |
| --- | --- | --- | --- | --- | --- |
| Table Analyzed | SNI vs HFTS | | | | |
| Two-way RM ANOVA | Matching: Across row | | | | |
| Assume sphericity? | Yes | | | | |
| Alpha | 0.05 | | | | |
| Source of Variation | % of total variation | P value | P value summary | Significant? |  |
| Row Factor x Column Factor | 0.461 | 0.995 | ns | No |  |
| **Row Factor** | **36.3** | **<0.0001** | ******** | **Yes** |  |
| **Column Factor** | **11.29** | **<0.0001** | ******** | **Yes** |  |
| Subject | 32.41 | 0.0567 | ns | No |  |
|  |  |  |  |  |  |
| ANOVA table | SS | DF | MS | F (DFn, DFd) | P value |
| Row Factor x Time | 33.33 | 7 | 4.762 | F (7, 40) = 0.1349 | P=0.9950 |
| Row Factor | 2625 | 7 | 375 | F (7, 40) = 6.399 | P<0.0001 |
| Time | 816.7 | 1 | 816.7 | F (1, 40) = 23.13 | P<0.0001 |
| Subject | 2344 | 40 | 58.59 | F (40, 40) = 1.659 | P=0.0567 |
| Residual | 1413 | 40 | 35.31 |  |  |
| Difference between column means |  | | | | |
| Mean of SNI | 18.49 | | | | |
| Mean of SNI+THz | 12.66 | | | | |
| Difference between means | 5.833 | | | | |
| SE of difference | 1.213 | | | | |
| 95% CI of difference | 3.382 to 8.285 | | | | |
| Data summary |  | | | | |
| Number of columns (Time) | 2 | | | | |
| Number of rows (Row Factor) | 8 | | | | |
| Number of subjects (Subject) | 48 | | | | |
| Number of missing values | 0 | | | | |

| Supplementary file 2c. The spike frequency in PYR of SNI mice before and after BLS | | | | | |
| --- | --- | --- | --- | --- | --- |
| Table Analyzed | SNI vs BLS | | | | |
| Two-way RM ANOVA | Matching: Across row | | | | |
| Assume sphericity? | Yes | | | | |
| Alpha | 0.05 | | | | |
| Source of Variation | % of total variation | P value | P value summary | Significant? |  |
| Row Factor x Column Factor | 1.416 | 0.9018 | ns | No |  |
| Row Factor | 11.29 | 0.0635 | ns | No |  |
| Column Factor | 0.07243 | 0.7101 | ns | No |  |
| Subject | 0.461 | 0.9716 | ns | No |  |
|  |  |  |  |  |  |
| ANOVA table | SS | DF | MS | F (DFn, DFd) | P value |
| Row Factor x Column Factor | 183.3 | 7 | 26.19 | F (7, 40) = 0.3915 | P=0.9018 |
| Row Factor | 4361 | 7 | 623 | F (7, 40) = 4.362 | P=0.0011 |
| Column Factor | 9.375 | 1 | 9.375 | F (1, 40) = 0.1401 | P=0.7101 |
| Subject | 5714 | 40 | 142.8 | F (40, 40) = 2.135 | P=0.0092 |
| Residual | 2676 | 40 | 66.9 |  |  |
| Difference between column means |  | | | | |
| Mean of SNI | 22.55 | | | | |
| Mean of SNI+Visible light | 21.93 | | | | |
| Difference between means | 0.625 | | | | |
| SE of difference | 1.67 | | | | |
| 95% CI of difference | -2.749 to 3.999 | | | | |
| Data summary |  | | | | |
| Number of columns (Column Factor) | 2 | | | | |
| Number of rows (Row Factor) | 8 | | | | |
| Number of subjects (Subject) | 48 | | | | |
| Number of missing values | 0 | | | | |

| Supplementary file 2d. The spike frequency in PYR of Sham mice before and after HFTS | | | | | |
| --- | --- | --- | --- | --- | --- |
| Table Analyzed | Sham vs HFTS | | | | |
| Two-way RM ANOVA | Matching: Across row | | | | |
| Assume sphericity? | Yes | | | | |
| Alpha | 0.05 | | | | |
| Source of Variation | % of total variation | P value | P value summary | Significant? |  |
| Row Factor x Column Factor | 4.831 | 0.0029 | ** | Yes |  |
| **Row Factor** | **66.5** | **<0.0001** | ******** | **Yes** |  |
| **Column Factor** | **15.75** | **<0.0001** | ******** | **Yes** |  |
| Subject | 5.691 | 0.7722 | ns | No |  |
| ANOVA table | SS | DF | MS | F (DFn, DFd) | P value |
| Row Factor x Column Factor | 342.6 | 7 | 48.95 | F (7, 40) = 3.824 | P=0.0029 |
| Row Factor | 4717 | 7 | 673.8 | F (7, 40) = 66.77 | P<0.0001 |
| Column Factor | 1117 | 1 | 1117 | F (1, 40) = 87.29 | P<0.0001 |
| Subject | 403.6 | 40 | 10.09 | F (40, 40) = 0.7884 | P=0.7722 |
| Residual | 512 | 40 | 12.8 |  |  |
| Difference between column means |  | | | | |
| Mean of Sham | 12.45 | | | | |
| Mean of Sham+HFST | 5.625 | | | | |
| Difference between means | 6.823 | | | | |
| SE of difference | 0.7303 | | | | |
| 95% CI of difference | 5.347 to 8.299 | | | | |
| Data summary |  | | | | |
| Number of columns (Column Factor) | 2 | | | | |
| Number of rows (Row Factor) | 8 | | | | |
| Number of subjects (Subject) | 48 | | | | |
| Number of missing values | 0 | | | | |

Note：PYR, pyramidal neurons; HFTS, high frequency terahertz stimulation; BLS, blue light stimulation
