# Supplementary material for: High-frequency terahertz stimulation alleviates neuropathic pain by inhibiting the pyramidal neuron activity in the anterior cingulate cortex of mice
Source: eLife. 2024 Sep 27;13:RP97444. doi: 10.7554/eLife.97444 (PMC11434610; doi:10.7554/eLife.97444)
Supplement: Source code 1. [file elife-97444-code1.zip › code/MClustV4.4Pro/MClust-4-4.docx]

MClust

Spike sorting toolbox

Documentation for version 4.4

Released December 2014.

Original MClust by

- *A. David Redish (ADR)*,
  current address, University of Minnesota, Minneapolis MN.

SPC (🡪 version 4.4) by

- *Brendan Hasz*

Major modifications that have been incorporated into versions 3.0-3.5 were made by

- *Neil Schmitzer-Torbert (NCST)*.
- *Jadin Jackson (JCJ)*.

Modifications that have been incorporated into version 3.0 were made by

- *Peter Lipa (PL)*.
- *Stephen Cowen (SLC)*.
- *Jadin Jackson (JCJ)*.
- *Francesco Battaglia (batta)*.

KlustaKwik (automated spike-sorter) was written by

- *Ken Harris*.

# Addition of SPC clustering to version 4.4

- We have added a new semi-automated clustering algorithm based on super-para-magnetic clustering (SPC) written by Brendan Hasz. It is very fast and does not need to be batch filed like KlustaKwik. Moreover, the .c code is included, so it can be easily mexed for any machine.

# Changes made to version 4.0

- The major changes for MClust 4.0 are to the underlying engine. The largest internal change is that everything is based on Packages and Objects. This should prevent some of the problems that have been arising from namespace overloading. However, this means that MClust is only compatible with Matlab versions >2012.
- Feature data file creation is now entirely done in memory. Theoretically, this means that one could have data files too large to analyze. However, with 64-bit matlabs, this shouldn’t be a problem.
- All calls are now based on correct capitalization.
- Cutters now maintain their own internal cluster lists. This means that clusters have to be exported to and imported from the primary MClust window.
- Cluster Quality has been integrated into the MClust process. You now have the option of writing .t, -wv.mat (waveform), and -cluQual.mat (cluster quality) files when writing .t files. There is also the option of writing “._t” files, which can be used to write data that you want to keep but may not want to use in all analyses (cut-off clusters, for example).
- The KlustaKwik selection window has changed dramatically. There are now three windows instead of one. (This is to match the shape of other cutters.) See description of KlustaKwik selection window below.
- If MClust finds itself in a strange state (which shouldn’t happen), you can reset it with ResetMClust typed in Matlab’s command line.

# Spike sorting

Neurophysiological recordings usually include spikes occurring on multiple cells simultaneously. It is important to be able to separate the spike trains of each of these cells. Because spikes occurring on different cells should show different waveform parameters (peak height, total energy, waveform shape, etc.), the spikes from a single cell will form clusters in that high-dimensional space (McNaughton, O’Keefe, and Barnes, 1983, *J. Neurosci. Methods*, 8:391–7; Fee, Mitra, and Kleinfeld, 1996, *J. Neurosci. Methods*, 76:3823–31). Tetrodes and stereotrodes have also proven useful for differentiating spikes from multiple cells: different cells show different spike shapes on each channel of the tetrode or stereotrode (McNaughton, O’Keefe, and Barnes, 1983, Wilson and McNaughton, 1993, *Science*, 261:1055–8). Many advances have been made in spike sorting in the last decade. There is not room to list those advances here. Where specific citations have been incorporated into MClust, those citations are noted in the text.

MClust is a toolbox which enables a user to perform automated and manual clustering on single‑electrode, stereotrode, and tetrode recordings. It allows manual corrections to automated clustering results. It outputs *t-files*, which contain (after a header) a list of timestamps in binary format. Timestamps are 32-bit longs at a resolution of 10 timestamps/ms. It is possible to modify the internal code to write timestamps as 64-bit longs. All data is stored internally as doubles in units of seconds.

## Requirements

MClust is written in Matlab™ (The MathWorks Inc., Natick MA) and requires Matlab™ version 2007a or higher. MClust-4.0 has been tested on PC workstations running the Windows (Microsoft Corp.) family of operating systems. It should, however, be portable to any system capable of running Matlab™ with an ANSI-compatible C++ compiler.

New components added in version 4.0 require toolboxes. If you do not have these toolboxes, you can still use MClust, but will not have the complete functionality. Specifically, the *Statistics toolbox* is needed for measuring Cluster Separation (see below). Warnings are given if the toolboxes are unavailable. These warnings can be suppressed with “warning off MCLUST:ToolboxUnavailable”.

## Disclaimer

This code is copyright © by the original authors (see above), 1998-2013.

None of the authors, nor their respective labs, nor their respective universities, assume any liabilities for this code. Use at your own risk. We have done our best to ensure that this code is correct, but do not make any guarantees. This program is distributed in the hope that it will be useful, but without any warranty; without even the implied warranty of merchantability or fitness for a particular purpose.

Use of this code should be acknowledged in any paper that uses data analyzed with it. Acknowledgement should be in the methods section as “(MClust-4.0, A. D. Redish et al)”. Use of automated cutters should be acknowledged as “KlustaKwik (K. Harris)”.

This code may be distributed freely. However, all distributed copies must include all components included in the original distribution. This code may not be modified without the express written consent of the author (contact A.D. Redish for permission).

[However, MClust is designed to facilitate the incorporation of new loading engines, new features and new cutting methods. If you have such that you wish to include, please contact me. - adr]

Send bug reports, questions, and comments to **redish@umn.edu**. Please do not send emails related to MClust to any of the other authors. None of the authors, nor their respective labs, nor their respective universities assume any responsibility for replying to such email, to fixing said bug-reports, or to maintaining this code. This code is distributed as is. [However, I will reply if I have time. I will do my best to fix bugs, answer questions, incorporate new features and cutters, etc. Many of the updates to this version were done by others, see above. - adr]

# Installing MClust

This assumes that you have already installed Matlab on your computer.

1. Create an *MClust* directory in the Matlab™ hierarchy.
2. Unzip the archive *(MClust-4.0.zip)* into the new *MClust* directory.
3. Add the *MClust* directory to your Matlab™ path (see Matlab™ information for how to do this). Make sure you add subdirectories.
4. Start up Matlab™ and type MClust and you’re off…

# Components

## Loading Engines

As of MClust-3.0 (and all subsequent versions), we have split out the loading engine. Any .dll or .m file placed in the *LoadingEngines* directory will be declared a plausible loading engine. In the Mclust main window (**Error! Reference source not found.**), there is a pop-up menu to select the loading engine.

### Loading engine requirements

A loading engine must be callable as a Matlab function. This means it must be either a .m function-file or a compiled mex function-file. It must take as input one or three inputs and provide one or two outputs. MClust-4.0 should work with any loading engine that supplies this functionality.

Loading engines must return data in Matlab double format. (This is the usual Matlab format for floating point numbers.)

The code should work with Loading Engines that return nChan > 4, but I have not tested it yet. (ADR)

#### INPUTS

- fn = file name string
- records_to_get = a range of values
- record_units = a flag taking one of 5 cases (1,2,3,4 or 5)

1. implies that records_to_get is a timestamp list.
2. implies that records_to_get is a record number list
3. implies that records_to_get is range of timestamps (a vector with 2 elements: a start and an end timestamp)
4. implies that records_to_get is a range of records (a vector with 2 elements: a start and an end record number)
5. asks to return the count of spikes (records_to_get should be [] in this case)

In addition, if only fn is passed in then the entire file should be read.

#### OUTPUT

- [t, wv]
- t = n x 1: timestamps of each spike in file
- wv = n x 4 x 32 waveforms

#### EXAMPLES

- [t,wv] = myLoadingEngine(‘myfile.dat’, 1:10, 2) should return the time and waveforms for the first 10 spikes in the file.
- t = myLoadingEngine(‘myfile.dat’) should return all the timestamps from the file.
- n = myLoadingEngine(‘myfile.dat’, [], 5) should return the number of spikes in the file.

In MClust-4.0.4 and higher, I have added a new LoadingEngine functionality. The code is backward compatible with old loading engines, but if a new loading engine provides for

- CV = LoadingEngine(‘get’, ‘ChannelValidity’);

where CV is a four-element logical vector, then the main MClust window will reset the four channel validity checkboxes to reflect the CV vector.

And

- XT = LoadingEngine(‘get’, ‘ExpectedExtension’);

where XT is a string (like ‘.ntt’ or ‘.nse’), then the expected extension of the data file will be reset to XT.

## Features

Features are processed parameters of spikes. Most features produce one number for each valid channel for each spike.

### Currently available features

energy — the energy contained within the waveform of each channel of the spike. Also known as the L2 norm. Produces one parameter for each valid channel. In the cutter, this feature will appear as e*nergy: Ch*.

peak — the maximum height of the waveform of each channel of the spike. Produces one parameter for each valid channel. In the cutter, this feature will appear as p*eak: Ch*.

time – the time of each spike.

wavePC1,2 — Returns for each waveform the contribution to the waveform that is due to the first (wavePC1) or second (wavePC2) principal component . In the cutter, this feature will appear as *wavePC1(or 2): Ch*.

### Extra Features

The following features are in the “Extra Features” directory. To add them to your feature list, copy them into the Features directory under MClust.

area — the area within the waveform of each channel of the spike. Area is defined as the integral of the positive component relative to zero plus the integral of the negative component relative to zero. Also known as the L1 norm. Produces one parameter for each valid channel. In the cutter, this feature will appear as a*rea: Ch*.

valley — the maximum depth of the waveform of each channel of the spike. Produces one parameter for each valid channel. In the cutter, this feature will appear as *Valley: Ch*.

peakValleyRatio — the ratio between the peak and valley parameters. Produces one parameter for each valid channel. In the cutter, this feature will appear as p*eakValleyRatio: Ch*.

spikeWidth — the width of each channel of each spike. Width is defined as the position (out of 32 samples) of the peak minus the position (out of 32 samples) of the valley. Thus *spikeWidth* can be negative. Since spikeWidth is integral, a small random value (±0.5) has been added to spikewidth to provide for scatter. This provides a better visual picture without changing the integrity of the spikeWidth parameter. In the cutter, this feature will appear as *spikeWidth: Ch*.

peakIndex — the sample at which the peak occurs in each spike; defined as the position (out of 32 samples) of the peak. Since peakIndex is integral, a small random value (±0.5) has been added to spikewidth to provide for scatter. This provides a better visual picture without changing the integrity of the peakIndex parameter. In the cutter, this feature will appear as *peakIndex: Ch*.

peak6to11 — the maximum height of the waveform of each channel of the spike in samples 6 to 11 of the wave form. Produces one parameter for each valid channel. In the cutter, this feature will appear as p*eak6to11: Ch*.

### Adding new features

Any Matlab function that is named “Feature_XXX.m” or “Feature_XXX.dll” and is placed in the *Features* directory will be included as a possible feature with the name XXX. Features must take as input three parameters and return as output three parameters. MClust-4.0 should work with any loading engine that supplies this functionality.

#### INPUT

- V = A tsd of tetrode data (time = n x 1 array of times, data = n x 4 x 32 array of waveforms)
- ttChannelValidity = nCh x 1 of Booleans
- Parameters = a cell array of internal information

#### OUTPUT

- FData = feature-calculated data (nSpikes x number of valid channels)
- FNames = a cell array of names (one for each valid channel)
- Parameters = a cell array of internal information. If the feature needs to be stopped and recalled, it will be repassed into the function as Parameters.

# Manual cutting

1. Start Matlab™.
2. Type MClust at the prompt. This will open the main MClust window (**Error! Reference source not found.**).
3. Select which features you wish to use to cluster the spikes. Features are moved between the *IgnoreFeature* and *UseFeature* lists by clicking on them.
4. Select the file to cut (you can select either the original data file or any feature data file from this file).
5. Click on ManualCut. This will open the Cluster Cutting Control Window (**Error! Reference source not found.**).
6. Cut your clusters.
   a. Click on *Redraw Axes* to open the Cutting Window.
   b. To add a cluster, select the *Add Cluster* button. When a cluster is added, it contains no boundaries.
   c. To add a boundary, under the functions selection menu for that cluster, select *Add points*. This will transfer the cursor to the cutting window. Select a set of points by clicking with the left button of the mouse. When you have selected the points, hit the enter key on your keyboard. For speed purposes, points are not drawn on the cutting window online. After hitting the enter key, the a boundary should appear. The boundary used is the convex hull of the points you selected. All spikes that fall within the boundary will change color to match that of the cluster, indicating that these spikes are now within that cluster. To add boundaries on other dimensions, change the axes drawn and add more limits. Spikes within a cluster are defined as those that fall within all of the boundaries defined for that cluster. **Change for MClust-4.0: there are now several different “add points” functions, “remove points” functions, etc. They should all be self-explanatory.**
   d. Continue adding clusters and boundaries (add boundaries with either inclusive or restrictive limits) until you are satisfied with the clusters.
   e. When you are done cutting clusters, exit the Cluster Cutting Control Window.
      **VERY IMPORTANT: In MClust-4.0, the clusters are stored separately in the Cluster Cutting Object. This means that you need to EXPORT the clusters to the main window or your clusters will be lost. You can do this by clicking the “export” button or by “Exiting (with Export)”. Append will add the clusters in your cutter to the end of the list in the main window. Overwrite will replace the clusters in the main window with the clusters in your**
7. Click on *Write Files* to save the processed clusters.
8. Exit MClust.

## Cutting clusters

The cluster cutting engine consists of up to three windows (**Error! Reference source not found.**). When the cutting engine starts up only the control window will be visible. To create a cutting window, click on *Redraw Axes*. Whenever the *Redraw Axes* checkbox is checked, any changes will be immediately shown in the cutting window. Unchecking and rechecking the *Redraw Axes* checkbox will redraw the cutting window.

Which 2D projection will be shown in the cutting window is controlled by the two selection boxes in the upper left corner of the control window. The arrows immediately below, steps through the possible projections. *View all dimensions* quickly steps through the projections.

Clusters are objects which define regions of the high-dimensional space. When the control window first opens, there will be no cutting clusters shown; only the *zero cluster* will be shown. The zero cluster is the set of all points. To add a cluster, select the *Add Cluster* button. Add clusters as necessary. Up to 99 clusters can be added.

When a cluster is added, it contains no points. To add points, under the *functions* selection menu for that cluster, select one of the *addpoints* methods. This will transfer the cursor to the cutting window. Select a region by clicking with the left button of the mouse. When you have selected this region, hit the enter key on your keyboard. For speed purposes, points defining the region are not drawn on the cutting window online. After hitting the enter key, a boundary should appear. The points included in the cluster will change color to match the color. To add boundaries on other dimensions, change the axes drawn and add more limits. Spikes within a cluster are defined as those that fall within all of the boundaries defined for that cluster.

You can exit and re-enter the cutting engine as many times as you want.

### Add points vs. Add limit

@mccluster type clusters in MClust 3.5 work by defining a set of points considered to be members of the cluster (using *Add points*), and refining that set of points by eliminating some of these cluster (using *Add limit*). Thus, the current members of a cluster are defined by the set of points that have been added that fall within the boundaries defined by the limits.

### Splitting a cluster

If you have a cluster that you wish to split into two clusters, copy the cluster and add new restrictive boundaries.

### Additional cluster features

Changing cluster color — Clicking on the color box next to the cluster will pop up a color control window allowing you to change the color used for the cluster. This color will be used for both boundaries and spikes falling within the cluster.

*Check cluster* — Shows key parameters for the cluster. Average waveform, ISI histogram, etc. See **Error! Reference source not found.**.

*Delete limit* — In the functions menu for each cluster, *Delete limit* removes the boundary for that cluster on the currently shown projection.

*Delete all limits* — In the functions menu for each cluster, *Delete all limits* removes all boundaries for that cluster.

*Delete all points* — Clusters created with KlustaKwik and BubbleClust do not have limits and must be deleted with *DeleteCluster*.

*Copy cluster* — In the functions menu for each cluster, *Copy cluster* creates a new cluster with the same boundaries as the cluster in question.

*Merge with* — In the functions menu for each cluster, *Merge with* asks for a cluster number and then creates a new cluster in which the boundaries are the convex hull of all the boundaries for the two clusters.

Hiding clusters — When the *Hide* checkbox next to a cluster is checked, no boundaries or spikes falling within that cluster will be drawn on the cutting window. The *Hide* and *Show* buttons on the left panel of the control window, hide or show all the clusters.

*Pack clusters* — Selecting the pack clusters button, removes all clusters that contain no spikes. Other clusters are moved up the list to fill the blank spaces.

*Undo* — MClust-3.5 has a ten-step undo.

### Final checks

After clusters have been cut, the clusters can be checked for key parameters using the following two buttons.

*CheckAllClusters* — Shows key parameters for each cluster currently defined (average waveform, interspike interval histogram, etc.).

*EvalOverlap* — Counts the number of spikes in each pair of currently defined clusters. The *Eval overlap* button pops up a window but also writes the table of overlaps to the Matlab™ control window. The formatting on the window can be misaligned, but the text output will always be correct.

### Cluster Separation

At a dinner meeting organized by Cliff Kentros at SFN*2002, it was noted that there was no standard technique for measuring recording quality with multi-channel electrodes (such as stereotrodes and tetrodes). At that meeting, it was noted that a measure of *cluster separation* would go a long way towards providing those quality measurements. MClust‑3.3 includes two measures *isolation distance* (developed by KD Harris *et al.* *Neuron,* 32:141, 2001) and *L-ratio* (developed by NC Schmitzer-Torbert *et al.* submitted‑a). Both measures assume that the cluster forms a multi-dimensional Gaussian in feature space. *Isolation distance* measures how many sigma one has to go to double the number of points in the cluster. *L-ratio* measures the number of extra-cluster points, weighted by the expected χ^2^ distribution expected from the Gaussian shaped cluster.

We have published a paper detailing these two measures and their respective properties (NC Schmitzer-Torbert *et al.* 2005). People wishing to report cluster separation values from MClust should cite this paper.

### ClusterOptions

**Each of the cluster types is now an object. Under the +MClust/ClusterTypes directory is a list of cluster types. Any function in the appropriate directory starting with the “ClusterFunc_” will be added as a method for that cluster and will appear in the list.**

These files will be visible in the functions list for each cluster. These include AverageWaveform, ISI histograms, etc.

Note: Any file that appears in the *ClusterOptions* directory will be available in the functions list for each cluster. Any file that takes as its sole input a cluster index (i.e. which cluster called it) and has no outputs can be used as an additional cluster option.

### MClustCutterOptions

**Each of the cutters are now an object. Under the +MClust/@CutterTYPE directory is a list of cluster methods. Any function in the appropriate directory starting with “CutterOption_” will be added as a method for that cutter and will appear in the list.**

### Autosave

MClust keeps track every time a change is made to the defined clusters. After 10 steps, it automatically saves the current clusters and the current defaults. The current clusters are saved in *autosave.clusters* and the current defaults are saved in *autodflts.mclust*. Clicking on the *Autosave* button forces an immediate autosave.

# Automated cutting with manual touch-up

*MClust-4.0* includes an automated spike-separation algorithm *KlustaKwik* (by Ken Harris). This algorithm finds clusters of points in a high-dimensional space. Because they work in all dimensions simultaneously, they tend to separate noise and real spikes better than manual cutting. However, this algorithm can also miss entire clusters as well as incorrectly merging two clusters. Therefore *MClust-4.0* offers the user the chance to touch-up and correct the automated algorithms.

KlustaKwik performs an expectation-maximization fit of *n* Gaussians to the data. This creates a set of putative clusters. As noted above, KlustaKwik will often split a single cell’s data into multiple clusters and may sometimes merge two clusters that are in fact separate cells. These can be corrected in the KlustaKwikDecisionWindow.

## SPC Cutter

MClust version 4.4 now includes a new automated spike clustering algorithm which is an approximation of superparamagnetic clustering (SPC). This algorithm finds clusters of points in high-dimensional space, and the hierarchical structure of those clusters. The SPC approximation algorithm creates a graph connecting all points, with edges between points which are close to one another (the superposition of the mutual K-nearest neighbor and minimum spanning graphs). Applying a threshold to the edges, such that edges longer than the threshold break, cause the graph to split into several subgraphs, which are the clusters. The algorithm finds the threshold values where splits occur, the corresponding clusters, and the split hierarchy.

The algorithm results in a hierarchy of clusters, and additional manual clustering should be done to “clean up the clustering”. A new cutter window allows the user to decide at what level of the hierarchy to assign clusters. The cluster hierarchy is displayed as a tree in the SPC cutter, and the user can decide which clusters to keep by navigating the tree and checking the boxes next to desired clusters. After exporting, these clusters can be edited in the manual cutter.

## KlustaKwik Decision Window

The KlustaKwik Decision Window has been extensively modified for MClust-4.0. In particular, it is now its own object, as are each of the clusters. **The most important change is that you will need to export clusters to the main window from the KlustaKwikDecisionWindow (KKDW) in order to access them in the manual cutting window.**

The structure of the KKDW has changed, but the general function has not.

First, the KKDW is a cutter subclass, just as the Manual Cutter is. Again, any method that is placed in its directory and starts with “CutterOption_” will appear as a method.

Second, the three windows (points display, waveforms, HistISI) have been separated so that they can be placed wherever you like for display.

Third, the buttons on the clusters have changed slightly. In order from left to right:

*Hide/Show* – as with any of the cutters, this hides or shows the data on the ClusterCuttingWindow.

*Color button* – as with any of the cutters, this changes the color of the dots displayed on the cutter window. All colors default to black.

*MergeName* – a string that defaults to KKxx, where xx is a number from 01 to 99. This string can be anything (you can type in this box). Any clusters with the same MergeName will be merged when exported. So if you want to merge two clusters, you can call them both “KK05” or “interneuron” or “bigcell” or whatever.

*Keep/toss toggle* – Clusters that are marked “keep” will be exported when exporting; clusters marked “toss” will not. All clusters default to “toss”.

*Focus radio button* – Only one cluster can have the focus at any time. The cluster with focus appears as blue larger dots in the dots display window, and shows the waveforms and HistISI in the other two windows (in blue). The cluster that has the focus will say “Focus”. All other clusters will show a number from 0 to 1, which is the correlation between their waveforms and the focus cluster.

*Comparison radio button* – Only one cluster can have the comparison at any time. When you change the focus, the comparison changes to match it. But while that cluster has the focus, you can select another cluster to compare to it. The cluster that has the comparison will be displayed as large red dots in the Cluster display window and will show red in the waveform and HistISI windows.

Generally the process is to click on focus, decide whether to keep/toss a cluster, look at the other clusters to see if they should be merged with the focus cluster. (You can use the comparison button to check the merge.) If you want to merge the two clusters, change the MergeNames to be the same. (Remember to KEEP both clusters.) Note, you can always merge later in the Manual Cutter, which has more powerful cutting and merge functions.

**Batch Processing**

MClust 3.0 supplied a program (RunClustBatch.m) that allowed the user to apply BubbleClust.exe or KlustaKwik.exe to data files. The output of these programs could then be viewed and refined using MClust 3.0. **MClust-4.0 has replaced this program with a function that takes parameters using the (‘parameter’, value) method. For example,**

RunClustBatch('minClusters', 10, 'maxClusters', 25);

RunClustBatch();

RunClustBatch('fcTT', {'TT1.ntt', 'TT2.ntt'});

The parameters are

% fcTT = {}; Pass in a cell array of filenames

% TText = '.ntt';

% StartingDirectory = pwd;

% FDDirectory = 'FD';

% channelValidity = true(4,1);

% LoadingEngine = 'LoadTT_NeuralynxNT';

% minClusters = 20;

% maxClusters = 60;

% maxSpikesBeforeSplit = []; % if isempty then don't split

% featuresToCompute = {'feature_Energy', 'feature_EnergyD1', 'feature_Peak', 'feature_WavePC1', 'feature_Time'};

% featuresToUse = {'feature_Energy', 'feature_EnergyD1'};

# Additional features

## Loading and saving defaults

The *defaults.mclust* file contains information for the way MClust looks. It saves the color scheme used for the clusters, the features used versus features ignored, the channel validity, and the loading engine.

Autosave saves the current default settings in *autodflts.mclust*. These can be loaded using the *Load defaults* button.

When MClust starts up it looks first for *defaults.mclust* in the current directory, then in the MClust directory.

### Loading, saving, clearing clusters

Clusters can also be loaded and saved separately. When loading clusters, the features used and the channel validity must be identical to when they were saved. *Write Files* saves clusters automatically.

Clearing clusters deletes all limits and packs the clusters. There is no undo for clearing clusters.

### Clearing the workspace

A button is supplied which clears all of the global variables used by MClust-4.0. Clicking this button is equivalent to exiting and restarting Matlab. It is recommended that the user click this button between cutting different data files (e.g., different tetrodes).

# Cutting non-tetrode data

Some people have used MClust to do spike sorting on single-electrode (SE) and stereotrode (ST) data. It should work on those data as well. In order to load SE or ST data, the user must (1) use an appropriate loading engine and (2) turn off the channel validity for channels 2, 3, & 4 (for SE) or 3 & 4 (for ST). Selecting an SE or ST loading engine does not automatically affect the channel validity.

A loading engine which loads SE or ST data must pad the waveforms with 0’s to fill out four channels. [We are looking into the possibility of making the code compatible with different size waveform matrices, but it turns out to be more complex than we hoped. – adr]

# FAQ (Troubleshooting / Warnings / Issues)

## Other platforms

“I have LINUX on my PC. Can I still run MClust?”

MClust has only been tested on PCs running the Windows family of operating systems. However, it should run on any machine that can run Matlab and has an ANSI‑compatible C++ compiler. If you want to try porting MClust to another platform, contact me at “redish@umn.edu” and I will do my best to help you.

## Matlab™ licenses

“Do I have to have Matlab™ to run MClust?”

Yes. MClust runs within the Matlab architecture. Therefore you will need to have Matlab to run MClust.

As noted above, some functionality requires toolbox licenses as well. If you do not have those toolboxes, MClust will still work and will still be able to cut clusters.
